# Publisher Correction: articles initially published in wrong volume

**DOI:** 10.1038/s41420-019-0186-2

**Published:** 2019-07-10

**Keywords:** Cellular neuroscience, Apoptosis

**Correction to: Cell Death Discovery**

10.1038/s41420-018-0065-2

10.1038/s41420-018-0066-1

10.1038/s41420-018-0067-0

10.1038/s41420-018-0068-z

10.1038/s41420-018-0069-y

10.1038/s41420-018-0070-5

10.1038/s41420-018-0071-4

10.1038/s41420-018-0072-3

10.1038/s41420-018-0073-2

10.1038/s41420-018-0074-1

10.1038/s41420-018-0075-0

10.1038/s41420-018-0076-z

10.1038/s41420-018-0077-y

10.1038/s41420-018-0078-x

10.1038/s41420-018-0079-9

10.1038/s41420-018-0080-3

10.1038/s41420-018-0081-2

10.1038/s41420-018-0082-1

10.1038/s41420-018-0083-0

10.1038/s41420-018-0084-z

10.1038/s41420-018-0085-y

10.1038/s41420-018-0086-x

10.1038/s41420-018-0087-9

10.1038/s41420-018-0088-8

10.1038/s41420-018-0089-7

10.1038/s41420-018-0090-1

10.1038/s41420-018-0091-0

10.1038/s41420-018-0092-z

10.1038/s41420-018-0093-y

10.1038/s41420-018-0094-x

10.1038/s41420-018-0095-9

10.1038/s41420-018-0096-8

10.1038/s41420-018-0097-7

10.1038/s41420-018-0098-6

10.1038/s41420-018-0100-3

10.1038/s41420-018-0101-2

10.1038/s41420-018-0104-z

10.1038/s41420-018-0102-1

10.1038/s41420-018-0103-0

10.1038/s41420-018-0105-y

10.1038/s41420-018-0106-x

10.1038/s41420-018-0107-9

10.1038/s41420-018-0108-8

10.1038/s41420-018-0109-7

10.1038/s41420-018-0111-0

10.1038/s41420-018-0112-z

10.1038/s41420-018-0110-1

10.1038/s41420-018-0113-y

10.1038/s41420-018-0114-x

10.1038/s41420-018-0115-9

Due to a technical error, content intended for publication in Volume 4 (2018) published in Volume 5 (2019). The content has been moved into the correct volume, and the citation information was updated accordingly.

